# Integrating Diet and Exercise for Effective Weight Management—Synergistic Strategies for a Complex Challenge

**DOI:** 10.3390/nu17213423

**Published:** 2025-10-31

**Authors:** Clemens Drenowatz, Klaus Greier

**Affiliations:** 1 Division of Sport, Physical Activity and Health, University of Education Upper Austria, 4020 Linz, Austria; 2 Department of Sports Science, Leopold-Franzens University Innsbruck, 6020 Innsbruck, Austria; nikolaus.greier@uibk.ac.at

Obesity and overweight, while largely preventable, remain among the most pressing public health challenges worldwide due to their adverse implications for cardiovascular disease, type 2 diabetes, musculoskeletal disorders, certain cancers, psychological well-being, and overall quality of life [1]. Despite decades of research and numerous intervention trials, progress in tackling the global rise in adiposity has been limited. The COVID-19 pandemic further exposed vulnerabilities in lifestyle behaviors, with many individuals becoming more sedentary and adopting less healthful diets, which exacerbated the risk for weight gain [2]. Global obesity rates have doubled among adults and quadrupled among children and adolescents between 1990 and 2022, resulting in more than one billion people living with obesity [3]. No country is currently on track to meet the global target of halting the rise in obesity, which also jeopardizes progress toward the goal of reducing premature mortality from noncommunicable diseases (NCDs) by one third by 2030 [4]. Consequently, the burden of disease attributable to overweight and obesity reached 128 million disability-adjusted life years (DALYs) in 2021, with an estimated total cost to health services of USD 990 billion per year, which represents over 13% of total healthcare expenditures [5,6]. In addition to direct medical costs, impaired productivity, disability, and reduced quality of life further elevate the socioeconomic burden associated with excess body weight [4].

While there has been considerable progress in the pharmacological treatment of obesity, behavioral choices remain a critical component in weight management. In fact, evidence-based guidelines on obesity medication recommend their use alongside reduced caloric intake and increased physical activity [7]. Furthermore, pharmacological treatment of obesity may not be affordable for the public health sector, and the use of expensive drugs to combat a lifestyle related problem should be questioned in general. Physical activity and exercise along with a healthy diet, on the other hand, are among the most cost-effective, high impact interventions [8]. Neither diet nor physical activity or exercise alone, however, offer a universal solution as positive aspects of a singular focus may be offset by compensatory adaptations. Beneficial effects achieved through exercise, for example, can be offset by compensatory increases in energy intake or sedentary behavior. Similarly, severe dietary restriction may lead to reductions in metabolic rate and spontaneous physical activity, undermining long term outcomes.

This Special Issue, therefore, explored dietary strategies and exercise or physical activity as interacting, co-determined systems. Contributions included randomized trials, observational studies, mechanistic explorations, and reviews, offering both clinical insight and research directions. In the following, two major themes that emerged from the contributions to this Special Issue are highlighted, along with considerations for future research.

**1.**  **Clinical Studies on Nutritional and Physical Interventions**

Prats-Arimon et al. demonstrated the benefits of a multimodal intervention targeting physical activity, diet, and mental health in adults with obesity (contribution 7). Following the nine-month intervention, nearly 90% of participants adopted an active lifestyle and better adherence to dietary recommendations, leading to significant improvements in body composition and metabolic risk profiles. Similarly, a clinical study among older adults with dynapenic obesity showed that combining resistance training with a moderate hypocaloric diet significantly enhanced body composition and strength (contribution 6). Interestingly, the addition of essential amino acid supplementation did not provide further benefits, suggesting that the fundamental components of diet and exercise already yield substantial health benefits. Lim et al., on the other hand, did not specifically examine the combined effects of diet and exercise and their study found that a healthy ketogenic diet led to more pronounced weight loss than standard caloric restriction (contribution 3). Despite the higher fat content of the ketogenic diet, improvements in metabolic profiles were also more marked, reinforcing the idea that the quality of dietary choices, rather than caloric restriction alone, is key to weight loss and overall health.

**2.**  **Considerations Beyond Energy Expenditure and Caloric Intake**

Among adolescents, Martínez-López and López-Gil reported that longer meal duration was associated with lower BMI z-scores, independent of physical activity and food consumption (contribution 4). These findings underscore that how we eat can influence body weight, suggesting that behavioral factors such as slower eating should be emphasized in interventions. Moreover, higher physical fitness has been shown to mitigate the negative effects of poor dietary habits in adolescents (contribution 2). Fitter youth were also more likely to make healthier dietary choices, which emphasizes the reciprocal relationship between exercise and nutrition. Particularly among children and adolescents, parents play a crucial role in the promotion of healthy eating behaviors and physical activity. Moliterno et al., however, found that parents frequently misperceive their child’s body weight, especially in children with overweight or obesity (contribution 5). Such a misperception may reduce their commitment to health promotion strategies. Improving weight recognition and awareness at the family level, therefore, should be incorporated into future intervention strategies.

Digital interventions also show promise, but conclusive evidence of their efficacy remains limited. A systematic review of eHealth and mHealth strategies in adults demonstrated beneficial effects on waist circumference, while effects on body composition and behavior change (e.g., diet, physical activity) were modest (contribution 1). The authors concluded that digital health tools should complement, rather than replace, traditional interventions. These findings highlight that implementation challenges, adherence, and contextual factors are as critical to intervention success as physiological mechanisms.

**3.**  **Future Directions**

The systematic review and studies included in this Special Issue further emphasize the heterogeneity of intervention outcomes, underlining the need for continued research. Despite advances in obesity management science, several gaps persist. Many interventions demonstrate short-term success but fail to follow up on weight loss maintenance or fail to induce sustainable weight loss maintenance. Furthermore, behavioral compensation remains poorly understood, even though such adaptations may explain individual differences in response to interventions. Physical activity may increase appetite or reduce non-exercise activity thermogenesis, while caloric restriction can suppress spontaneous movement. Thus, personalized approaches that integrate psychological, environmental, genetic, and microbiome factors are essential. Translating interventions across diverse cultural, socioeconomic, and healthcare contexts will also require systems thinking and translational research. Although emerging technologies such as AI-guided coaching and precision nutrition may offer tools to tackle these challenges, they must be rigorously validated in real-world conditions before large-scale adoption.
